# Investigating long-term health outcomes in high-level rugby union players using linked administrative data

**DOI:** 10.23889/ijpds.v9i5.2797

**Published:** 2024-09-18

**Authors:** Stephanie D'Souza, Barry J. Milne, Francesca Anns, Kenneth L. Quarrie

**Affiliations:** 1 The University of Auckland; 2 New Zealand Rugby

## Objective and Approach

The Kumanu Tāngata study investigates long-term health outcomes in male high-level rugby union players in New Zealand using the Integrated Data Infrastructure (IDI), a comprehensive de-identified whole-population administrative research database. A historical register containing players active between 1950-2000 was linked to the IDI. Rugby players (n=12,861) were compared to general population males (n=2,385,690), matched by age, ethnicity, and birthplace, on neurodegenerative disease outcomes from health records available between 1988-2018. Cox proportional hazards models were conducted.

## Results

Rugby players exhibited a higher neurodegenerative disease incidence (4.7%) than the general population (3.9%). Players had an increased risk of any neurodegenerative disease (1.17 [95% CI 1.08–1.27]), Alzheimer's disease (1.42 [95% CI 1.22–1.66]), and other dementias (1.16 [95% CI 1.05–1.27]). An increased risk of any neurodegenerative disease was observed for professional- or international-level play (HR 1.51 [95% CI 1.04–2.18]), six or more years of play (HR 1.32 [95% CI 1.15–1.50], and a backline playing position (HR 1.30 [95% CI 1.17–1.45]).

## Conclusions

The study indicates a small-to-moderate increased risk of neurodegenerative diseases for high-level rugby players in New Zealand when compared to the general population, with variations observed across play duration, level and position. Future research will examine additional health outcomes, including mortality, musculoskeletal conditions, and mental health disorders.

## Implications

This large-scale linkage study allows for a comprehensive examination of long-term health outcomes and enhanced statistical power than previous research. Findings have the potential to influence health policies and athlete care strategies in contact sports.

